# Multiple routes to help you roam: A comparison of training interventions to improve cognitive-motor dual-tasking in healthy older adults

**DOI:** 10.3389/fnagi.2022.710958

**Published:** 2022-11-03

**Authors:** Rachel Downey, Louis Bherer, Kristell Pothier, Tudor Vrinceanu, Brittany Intzandt, Nicolas Berryman, Maxime Lussier, Thomas Vincent, Antony D. Karelis, Anil Nigam, Thien Tuong Minh Vu, Laurent Bosquet, Karen Z. H. Li

**Affiliations:** ^1^Department of Psychology, Concordia University, Montréal, QC, Canada; ^2^PERFORM Centre, Concordia University, Montréal, QC, Canada; ^3^Département de Médecine, Université de Montréal, Montréal, QC, Canada; ^4^Centre de recherche de l’Institut de cardiologie de Montréal, Montréal, QC, Canada; ^5^Centre de recherche de l’Institut universitaire de gériatrie de Montréal, Montréal, QC, Canada; ^6^Psychologie des Ages de la Vie et Adaptation, University of Tours, Tours, France; ^7^Département des sciences de l’activité physique, Université du Québec à Montréal, Montréal, QC, Canada; ^8^Centre hospitalier Université de Montréal, Montréal, QC, Canada; ^9^Faculté des sciences du sport, Université de Poitiers, Poitiers, France

**Keywords:** aging, executive function, gait, dual-task, exercise, cognitive training

## Abstract

Cognitive-motor dual-tasking is a complex activity that predicts falls risk and cognitive impairment in older adults. Cognitive and physical training can both lead to improvements in dual-tasking; however, less is known about what mechanisms underlie these changes. To investigate this, 33 healthy older adults were randomized to one of three training arms: Executive function (EF; *n* = 10), Aerobic Exercise (AE; *n* = 10), Gross Motor Abilities (GMA; *n* = 13) over 12 weeks (1 h, 3×/week). Single and dual-task performance (gait speed, m/s; cognitive accuracy, %) was evaluated before and after training, using the 2-back as concurrent cognitive load. Training arms were designed to improve cognitive and motor functioning, through different mechanisms (i.e., executive functioning – EF, cardiorespiratory fitness – CRF, and energy cost of walking – ECW). Compared to baseline, we observed few changes in dual-task gait speed following training (small effect). However, dual-task cognitive accuracy improved significantly, becoming facilitated by walking (large effect). There were no differences in the magnitude of improvements across training arms. We also found that older adults with lower cognitive ability (i.e., MoCA score < 26; *n* = 14) improved more on the dual-task cognitive accuracy following training, compared to older adults with higher cognitive ability (i.e., MoCA ≥26; *n* = 18). Taken together, the results suggest that regardless of the type of intervention, training appears to strengthen cognitive efficiency during dual-tasking, particularly for older adults with lower baseline cognitive status. These gains appear to occur via different mechanisms depending on the form of intervention. Implications of this research are paramount, as we demonstrate multiple routes for improving cognitive-motor dual-tasking in older adults, which may help reduce risk of cognitive impairment.

## Introduction

Cognitive-motor dual-task performance (e.g., walking while talking) in older adults has been shown to predict future physical and cognitive decline, including mild cognitive impairment and dementia (Montero-Odasso et al., 2012). Cognitive-motor dual-tasking is a multi-faceted behavior, involving cognitive processes, particularly executive functions (EFs), as well as motor skills. The complex interaction between cognitive and motor domains helps explain why different forms of cognitive or physical interventions have been found to maintain or improve dual-task performance in healthy older adults (Berryman et al., 2014; Wollesen and Voelcker-Rehage, 2014; Fraser et al., 2017). Nevertheless, the potential mechanisms which underlie improvements in dual-task performance across different training modalities are less well-understood. Therefore, the primary aim of this study was to compare the improvements of three different interventions on dual-task performance in healthy older adults and to investigate the mechanisms that could explain these improvements.

As individuals grow older, there is a greater reliance on cognitive resources, particularly EFs, when completing a motor task, such as walking (Hausdorff et al., 2005). This is well-supported by dual-task experiments, wherein dual-task costs (DTCs), or the decrement observed during dual-compared to single-task performance, are found to be greater in older adults compared to younger adults (eg., Li et al., 2001). This decrement in performance has also been shown to occur in older adults at lower levels of cognitive load (Srygley et al., 2009), and with increased physical task complexity (e.g., usual vs. fast paced walking; Krampe et al., 2011), compared to younger adults. This suggests that with aging, fewer cognitive resources are available to allocate attention to a secondary task. Indeed, neuroimaging evidence shows increased prefrontal cortex activity during dual-task compared to single-task walking, suggesting that more cognitive resources are required for complex gait (Holtzer et al., 2014; Mirelman et al., 2017).

Moreover, according to the posture-first hypothesis, when given instructions to equally prioritize both the motor and cognitive task during dual-tasking, older adults prioritize walking, showing greater DTCs in the cognitive domain, while younger adults show more even emphasis across tasks (Li et al., 2001; Verghese et al., 2007). This asymmetry might be due to the greater survival value attributed to walking in old age, thereby leading to greater priority, as compared to the simultaneous cognitive task performance. Together, these findings suggest that age-related declines in cognitive capacity play an increasing role in gait with aging. Interventions aimed at enhancing cognitive capacity in older adults may therefore be critical for improving cognitive-motor dual-tasking.

The extant literature suggests that computerized EF training leads to near-transfer effects, such as inhibitory control, divided attention, and task-switching (Desjardins-Crepeau et al., 2016; Bherer et al., 2021a) as well as far-transfer effects including dual-task balancing (Li et al., 2010; Smith-Ray et al., 2015) and walking (Verghese et al., 2010; Smith-Ray et al., 2015; Fraser et al., 2017). Such improvements to motor performance after cognitive training are attributed to the increase in cognitive resources available for dividing one’s attention between the cognitive and motor tasks (Li et al., 2018).

Aerobic exercise (AE) has also been associated with enhanced EF (e.g., inhibition and working memory; Colcombe and Kramer, 2003), brain plasticity (Erickson and Kramer, 2009; Weinstein et al., 2012), and dual-task walking and balance (Fraser et al., 2017). Stillman et al. (2016) propose a conceptual model for possible mechanisms of physical activity in mediating neurocognitive functioning, including cellular and molecular changes, which initiate macroscopic changes to the brain and behavior, that, in turn, influence cognition. The cardiovascular hypothesis, which is one component of the model, suggests that aerobic capacity or cardiovascular efficiency may mediate improvements in executive functioning, which could increase cognitive resources required during dual-task processing (Stillman et al., 2016). Indeed, recent research has shown that in older adults, increased cardiorespiratory fitness mediated the improvements seen in processing speed for older-old adults, and task-set costs (i.e., the ability to maintain different response alternatives in memory and prepare to answer multiple tasks) in younger-old adults following AE (Bherer et al., 2021b). Moreover, increased cardiorespiratory fitness may improve dual-task walking performance by decreasing the relative intensity of the walking task for a given gait speed. This, in turn, may reduce the demands of executive control during dual-tasking and allow more attention to be allocated to the cognitive task.

Gross motor abilities training (GMA), also termed *coordination training*, has shown far transfer effects in improving cognitive processes such as executive control and processing speed, as well as decreasing prefrontal activity, suggesting more efficient information processing (Voelcker-Rehage et al., 2011). GMA training has also been shown to improve inhibitory control under single (Forte et al., 2013) and dual-task conditions, as well as maximum walking speed (Berryman et al., 2014). According to this framework, gait impairments observed in older adults are suggested to be due in part to altered coordination, which increases the amount of energy required to walk due to motor inefficiency (Van Swearingen and Studenski, 2014). Therefore, improvements in dual-task performance following GMA training are suggested to be due to increased coordinated walking abilities, thereby reducing the energy cost of walking (ECW). In turn, this would reduce the relative intensity of the walking task and allow for more resources to be allocated to the cognitive domain. Together, this research suggests that EF, AE, and GMA training have a strong potential to improve cognitive-motor dual-task performance, which may be mediated by different underlying mechanisms.

As previously mentioned, comparisons of interventions on cognitive-motor dual-task performance in older adults show similar improvements across groups, providing evidence for a multiple routes perspective. Specifically, Berryman et al. (2014) found similar improvements in cognitive performance during a dual-task condition following either combined resistance with AE training or GMA training. Moreover, in a study contrasting different combinations of active training conditions (EF, AE) and active control conditions (computer lessons, stretching), comparable dual-task improvements were found across the active training groups, including dual-task walking speed, balance (Fraser et al., 2017), and functional mobility (Desjardins-Crepeau et al., 2016). Finally, Pothier et al. (2021) found that global mobility (i.e., Timed up and Go) improved to a similar extent in older adults following either EF, AE, or GMA training. However, no research to date has compared the effect of these three well-established training approaches on cognitive-motor dual-task performance in healthy older adults.

In summary, older adults have poorer dual-task performance compared to younger adults, which is in part due to reduced cognitive resources and motor alterations (Van Swearingen and Studenski, 2014; Stillman et al., 2016; Li et al., 2018). Although there is substantial evidence to suggest that certain interventions may help maintain or improve dual-task performance in older adults, there is limited research directly comparing EF, AE, and GMA training. We therefore sought to investigate the effects of (i) EF (ii) AE, and (iii) GMA training on cognitive-motor dual-tasking in older adults and to examine the mechanisms underlying each intervention using a proof of concept study design.

We hypothesized that (1) following training, there would be a greater reduction in cognitive dual-task costs (DTCs) compared to gait DTCs. Based upon the posture-first hypothesis, that older adults tend to exhibit greater DTCs to cognitive performance than to walking (Li et al., 2001), there is greater potential for cognitive improvement than motor improvement. We also anticipated that (2) all participants, regardless of the training arm, would have similar improvements in cognitive DTCs following training, based upon previous research findings of null group differences on dual-task costs (i.e., 3). In order to verify the intended underlying mechanisms of the interventions, we hypothesized that (3) the different training arms would lead to specific improvements in: (i) EF following EF training, (ii) cardiorespiratory fitness (i.e., VO_2peak_) following AE, and (iii) ECW following GMA. This hypothesis was grounded in research on the proposed mechanisms of each intervention (Van Swearingen and Studenski, 2014; Li et al., 2018; Bherer et al., 2021b), as well as a joint study from our laboratory that used the same training design, but assessed functioning mobility rather than cognitive-motor dual-tasking (Pothier et al., 2021).

## Materials and methods

### Participants

**Figure 1:**
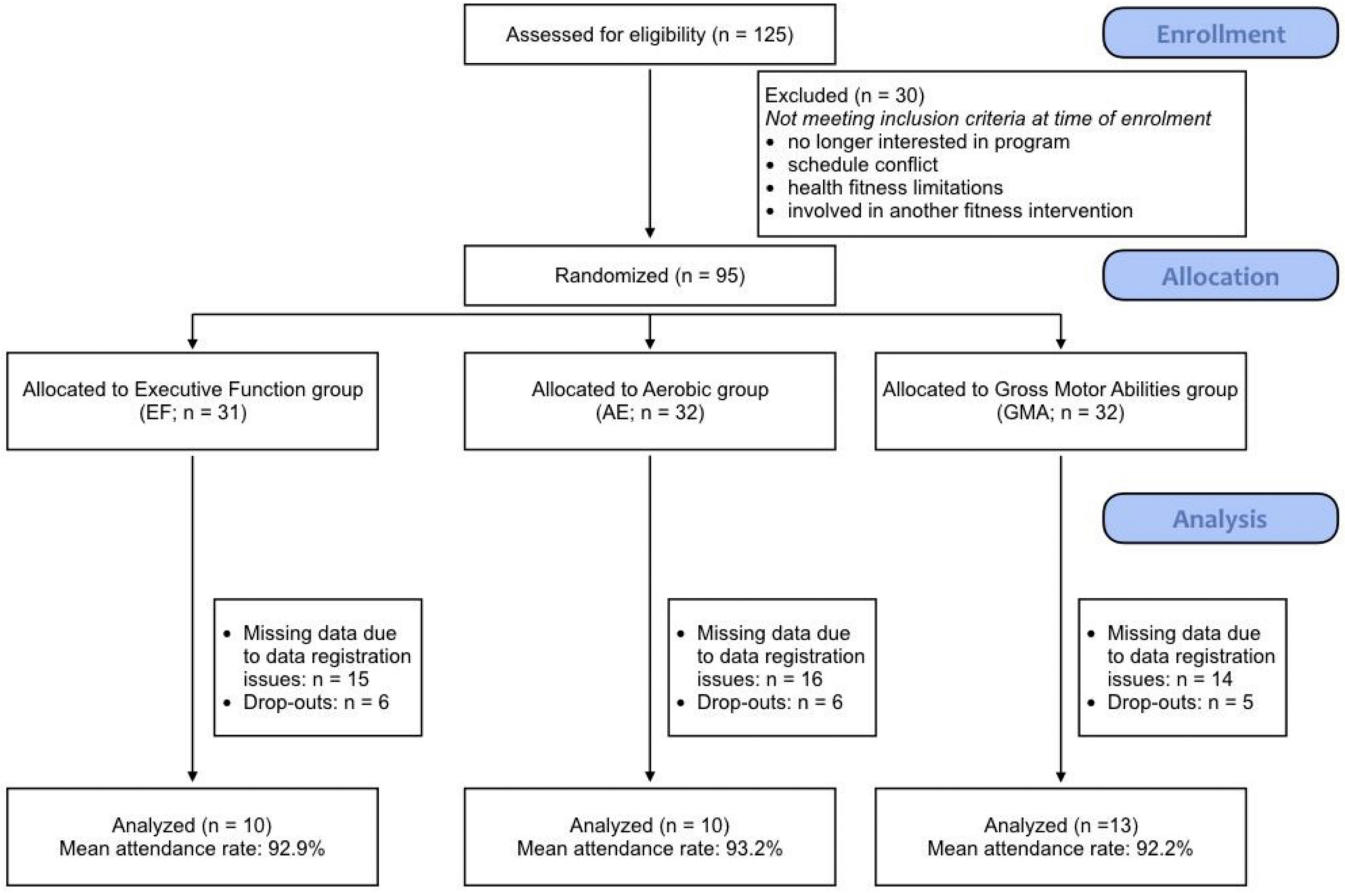
CONSORT flow diagram. Note: The final row depicts the number of participants included in the study.

A total of 125 community-dwelling older adults were first recruited and assessed for eligibility. Thirty participants did not meet inclusion criteria at the time of enrollment, resulting in 95 participants being randomized to each of the three training arms. A total of 17 participants abandoned the intervention voluntarily before its completion (6 from EF: study too demanding = 1, sickness/health issues = 2, no longer interested = 2, too many absences = 1; 6 from AE: study too demanding = 1, sickness/health issues = 2, no longer interested = 3; 5 from GMA: study too demanding = 1, sickness/health issues = 2, no longer interested = 1, involved in another parallel study = 1). Due to data-registration issues concerning the dual-task data for the first six cohorts, we have opted to report only the data for the subsequent cohorts after the problem was addressed. This resulted in a subset of 33 participants (EF = 10, AE = 10, GMA = 13) that were included in the present analyses (see Figure 1 for CONSORT diagram). There were no significant differences between groups in terms of attendance and drop-out rates, suggesting similar adherence across groups. There were also no significant differences in the participant characteristics between participants who had complete data and were analyzed for this study compared to participants who had missing data (see Table 1). Finally, results from Little’s MCAR test showed that the data were missing completely at random, χ^2^(21) = 23.40, *p* = 0.323. These findings suggest that the subset of participants is representative of the full dataset and that the data are not biased.

**Table 1:** Dual-task costs before and after training across groups.

		Cognitive DTC (%)			Walking DTC (%)		
		Mean (SD)	*F*	*p*	Mean (SD)	*F*	*p*
Between-subjects effects							
Group	EF	0.90 (9.77)	4.36	0.022*	−2.29 (7.41)	0.928	0.839
	AE	2.96 (9.77)			1.62 (7.41)		
	GMA	−8.22 (9.77)			1.47 (7.41)		
							
Within-subjects effects							
Time	Pre-training	3.89 (12.8)	8.82	0.006*	0.917 (7.45)	0.618	0.438
	Post-training	−8.03 (17.5)			−0.16 (10.1)		
Time x Group	EF Pre-training	7.49 (15.5)	0.169	0.845	−1.38 (8.18)	0.911	0.413
	EF Post-training	−5.70 (11.7)			−3.20 (9.55)		
	AE Pre-training	7.19 (10.5)			3.53 (8.36)		
	AE Post-training	−1.27 (14.1)			−0.30 (11.0)		
	GMA Pre-training	−1.41 (11.2)			0.67 (5.95)		
	GMA Post-training	−15.0 (21.8)			2.27 (9.90)		

DTC = dual-task cost as derived by [(ST – DT)/ST] * 100. There was a significant reduction in cognitive DTCs following training (negative score indicates dual-task facilitation, such that cognitive accuracy was better during dual-tasking, compared to single-tasking), with no significant change in walking DTCs following training.

A statistical power analysis was performed for sample size estimation, based on published data (Fraser et al., 2017) which compared n-back accuracy and walking speed before and after 12 weeks of different combinations of AE, EF, and placebo controls. The effect sizes for the main effect of Time in this study (n-back accuracy: *n^2^* = 0.11; walking speed: *n*^2^ = 0.29) are considered to be large using Cohen’s (Cohen, 1988) criteria. With an alpha set at.05 and power set at 0.95, the projected sample size needed to find a significant main effect of Time with this effect size (G*Power 3.1) is approximately n = 30. Accounting for possible attrition (20%), a total of n = 36 participants is required. Thus, our final sample size of n = 33 is adequate for the main objective of this study. We also provide effect sizes to support the strength of the observed findings. Nevertheless, we recognize that the small sample size due to data-registration issues is a limitation of the study.

Eligible participants were 60 years or older, able to speak fluently, and comprehend either English or French, and were not on medications that could impair their cognitive and physical test performance. Participants were excluded if they participated in a structured training program over the last year, failed the assessment of readiness to exercise (PAR-Q+; Thomas et al., 1992), or had a chronic medical condition such as cardiopulmonary or musculoskeletal diseases, neurological disease, or early signs of dementia (<26 on the Mini-Mental State Exam: MMSE; Folstein et al., 1983), depression (≥11 Geriatric Depression Scale; Brink et al., 2013), or major uncorrected perceptual limitations. Participant characteristics by treatment group are shown in Table 2. All participants provided informed consent as approved by the Institut Universitaire Gériatrie de Montreal Ethical Research Committee and Concordia University’s Human Research Ethics Committee.

**Table 2:** Demographic information and baseline cognitive and physical capacity across training groups for participants included in the statistical analyses and participants who were not due to missing data.

Characteristic	Analyzed data			Missing data		
	EF	AE	GMA	EF	AE	GMA
	*n* = 10	*n* = 10	*n* = 13	*n* = 16	*n* = 14	*n* = 14
Age (years)	70.0 (6.09)	68.2 (5.34)	70.2 (3.85)	70.4 (5.43)	69.1 (4.49)	70.7 (5.30)
Sex (*n*, % male)	7 (70.0)	4 (40.0)	5 (38.5)	5 (29.4)	3 (20.0)	5 (31.3)
Education (years)	16.2 (2.70)	18.0 (4.55)	17.4 (5.30)	15.1 (4.31)	15.29 (2.64)	15.0 (2.16)
MoCA	25.4 (2.55)	27.7 (1.89)	25.2 (2.66)	26.4 (2.72)	26.6 (2.59)	26.4 (3.45)
MMSE	28.3 (1.51)	28.5 (1.58)	28.7 (1.21)	28.4 (0.84)	28.7 (1.27)	28.3 (1.50)
TUG (sec.)	9.18 (1.34)	7.79 (1.36)	9.20 (1.40)	9.36 (1.51)	8.80 (1.32)	9.30 (1.86)

MoCA = Montreal Cognitive Assessment, MMSE = Mini-Mental State Exam, TUG = Timed Up and Go. Values indicate means and standard deviations in brackets. There were no significant differences across training groups, suggesting that randomization was appropriate. There were no significant differences between participants included in the main statistical analyses and those who were not due to missing data, suggesting that the participant characteristics of the smaller dataset are representative of the full dataset.

### Procedure

Participants were first screened for eligibility with a phone interview and then a medical evaluation by a geriatrician. Eligible participants completed a pre-test evaluation of cognitive and physical measures, including the dual-task assessment. Participants were then randomized into one of three training protocols: EF, AE, or GMA, which consisted of three, 1-h sessions a week, for 12 weeks (completed in small groups of 5–8 individuals). Within 3 weeks after training, participants completed a post-test evaluation using the same measures.

### Measures

In addition to the tests described below, other outcome measures (e.g., global mobility, neuropsychological functioning) were administered to the larger sample and are presented elsewhere (Pothier et al., 2021; Vrinceanu et al., 2022).

### Background measures

Cognitive functioning was screened using the MMSE (/30; Folstein et al., 1983; Brink et al., 2013) and Montreal Cognitive Assessment (MoCA; /30; Nasreddine et al., 2005; Brink et al., 2013). While a MoCA score below 26 can be indicative of mild cognitive impairment, participants who scored below this were still included if they performed above the clinical cut-off on the MMSE (i.e., 26). Global mobility was also assessed using the Timed Up and Go (TUG) task (Shumway-Cook et al., 2000).

### Primary outcome measures

#### Cognitive task

An auditory 2-back task (Fraser et al., 2017) served as the cognitive outcome measure in single- and dual-task conditions. In the single-task condition, participants performed the 2-back task while standing. In the dual-task condition, they performed the task while walking (see below). Randomly ordered single digits were presented through a set of wireless headphones (Sennheiser Canada, Pointe-Claire, QC, Canada) at a 2-s rate, for a total of 30 s. Participants were asked to recall out loud the number they heard two items previously (2-back). The responses were manually recorded during each trial, then converted to accuracy scores (% correct out of 15). *Walking Task.* Participants were instructed to walk around a 23-meter oval track, demarcated by a single stretch of tape on the floor, at their normal walking speed. Each walking trial was initiated by the audio signal, “GO,” heard through wireless headphones, and ended with the audio signal, “STOP.” Each trial lasted 30 s. The distance walked was manually recorded and divided by 30 s to attain a measure of gait speed (m/s). *Dual-Task.* Participants completed the 2-back cognitive task while concurrently walking at a self-selected pace. They were instructed to perform both the cognitive and walking task equally well.

Participants were first familiarized with each of the single tasks before introducing the dual-task procedure. Feedback on performance was only given during the familiarization portion of the task. Each block consisted of three trials (single-task 2-back, single-task walk, and dual-task), which were repeated across four blocks. Dual-task costs (DTC; %) were calculated as: [(single-task – dual-task)/single task * 100], for cognitive accuracy (i.e., cognitive DTCs) and gait speed (i.e., walking DTCs; greater positive number indicates poorer performance when completing the dual-task compared to single-task). DTC change scores were calculated by subtracting the post-training DTCs from the pre-training DTCs (greater negative number indicates more improvement following training).

### Secondary outcome measures

In addition to the primary experimental outcomes, three indexes were included to address the underlying mechanisms associated with each training type and assessed during pre-and post-training phases so that the *magnitude of change* in executive function, aerobic capacity, and motor skills could be quantified and considered as potential predictors of change in dual-task walking.

#### Changes in executive function

Changes in executive functions due to training were measured using a variant of the Stroop task used during training. Instead of using letters, the pre-and post-intervention variant involved digits. The assessment comprised five different conditions (familiarization, reading, counting, inhibition, and switching); however, reaction times for the inhibition and switching trials were analyzed as potential mechanisms underlying the EF training as these tasks rely most heavily on executive function. In the inhibition condition, digits were presented on the screen, whereby the number of digits was incongruent with the digit displayed (e.g., five number twos were presented). Participants were instructed to identify the quantity of digits presented, while inhibiting responses indicating the digits that were displayed on the screen. In the switching condition, the stimuli were identical to those in the inhibition condition, except that on some trials, the digits were surrounded by a white frame, to indicate a goal switch, whereby participants had to identify the digit that was presented on the screen, rather than indicating the quantity of digits.

#### Changes in cardiorespiratory fitness

The proposed mechanism thought to underlie the AE training protocol was cardiorespiratory fitness, as measured by peak oxygen uptake (VO_2peak_). The detailed protocol has been described previously (Berryman et al., 2013). Briefly, participants wore an electrocardiogram to monitor heart rate and a mask to measure gas exchange during a maximal graded exercise test on a cycle ergometer. The test began at a pre-defined load and then increased in workload by 15 Watts. Testing was completed when participants reached exhaustion (i.e., were unable to maintain the cadence of 60 to 80 revolutions per minute) or according to reasons described by the ASCM (American College of Sports Medicine, 2001). VO_2Peak_ was defined as the highest volume of oxygen consumed over a 30 s interval in ml.kg^−1^.min^−1^.

#### Change in the energy cost of walking

The mechanism thought to underlie the GMA training protocol is the energy cost of walking (ECW). All participants were equipped with the same mask to measure the O_2_ consumption and CO_2_ production as during the VO_2Peak_ assessment. However, participants walked on the treadmill during 6 min at a constant speed of 4 km.h^−1^. The oxygen cost of walking (OCW), in ml.kg^−1^.min^−1^, represents the mean VO_2_ from the last 2 min of the walking task. The ECW was calculated as described elsewhere (Berryman et al., 2012). Briefly, the gross OCW (ml.kg^−1^.min^−1^) was divided by the walking speed (m.min^−1^) to obtain a value in ml.kg^−1^.m^−1^. Thereafter, values in ml.kg^−1^.m^−1^ were first converted into L.kg^−1^.m^−1^. Using the respiratory exchange ratio (RER) corresponding to the last 2 min of walking, an appropriate energy equivalent of oxygen (J.L^−1^) was used to convert the previously calculated ECW (L.kg^−1^.m^−1^) in J.kg^−1^.m^−1^. RER had to be below 1 during the last 2 min of walking so that oxygen values could be considered for further analyses. These procedures are in agreement with the scientific literature for moderate-intensity exercise (Xu and Rhodes, 1999; Fletcher et al., 2009).

### Training protocol

#### Executive function training

The EF intervention was completed on individual tablets while seated. Participants completed three executive function tasks per session: (i) visual n-back, (ii) Stroop, and (iii) dual-task (20 min/task).

The n-back task was designed to improve updating. Participants were required to indicate whether the current number presented matched the number from *n steps* earlier in the sequence (Kirchner, 1958). The stimuli included numbers between “1” to “9.” Reaction times (ms) and accuracy (total number correct/maximum possible correct) were recorded. Difficulty levels were incremented over the 3 months of training (from 1-back to 3-back).

The modified Stroop task was designed to improve inhibition and switching and was comprised of five different conditions (familiarization, reading, counting, inhibition, and switching). In the familiarization condition, participants were required to press a button corresponding to the digit presented on the screen (“1” to “3” with their left thumb, “4” to “6” with their right thumb). In the reading condition, multiple identical digits were presented in a small group, where the identity of the digit corresponded with the quantity of the digits presented (e. g., four copies of the digit “4”), and participants had to press the corresponding button with their thumb. In the counting condition, groups of one to six asterisks were presented and the participants had to report how many asterisks were present. In the inhibition condition, letters were presented in small identical groups; however, the letters presented were incongruent with the larger letter that was formed (e.g., copies of small letters “L” to form a big “H”). Participants were instructed to identify the larger formed letter, while avoiding responses to the small grouped letters. In the switching condition, the stimuli were identical to those in the inhibition condition, except that on some trials, the group of small letters was surrounded by a white frame, to indicate a goal switch, whereby participants had to identify the small letters instead of the bigger formed letter for those trials only.

The dual-task program was designed to improve divided attention by having participants perform two discrimination tasks either alone or simultaneously. Stimuli were presented either visually (e.g., fruits vs. modes of transport; letters vs. numbers) or orally (sounds vs. beeps) using headphones. Participants completed blocks of single-task trials (Pure blocks) or mixed trials that randomly involved one or both tasks (Single-mixed trials and Dual-mixed trials, respectively). For Dual-mixed trials, participants were instructed to respond to both stimuli equally. However, after two training sessions, participants were encouraged to prioritize one hand over the other to increase the level of difficulty (i.e., in the dual-mixed trials when two stimuli were presented, participants were asked to make a response using their left or right hand first before making a response with the other hand).

#### Aerobic exercise training

The AE training involved recumbent cycling designed to enhance cardiorespiratory fitness and aerobic endurance. Each training session alternated between high-intensity interval exercises and moderate-intensity continuous exercises. Such a program was previously implemented and led to significant improvements in cardiorespiratory fitness (Berryman et al., 2014). Maximal Aerobic Power (MAP) was measured at baseline and represents the highest mechanical power output (Watts) produced by participants at the end of a maximal graded test performed on a cycle ergometer (Lode, CORIVAL). Participants began at a pre-defined starting workload for women (35 Watts) and men (50 Watts). The workload then increased by 15 Watts until exhaustion. Participants were required to maintain a pedaling rate of 60 to 80 revolutions per minute. Testing was completed when participants were unable to maintain the cadence or according to reasons described by the American College of Sports Medicine (American College of Sports Medicine, 2001). This information was used to calibrate individualized workload for the AE training.

Each training session included a 10-min warm-up, during which participants maintained 50% of their MAP. For the HIIT sessions, the warm-up was followed by two 5-min intervals, during which participants alternated between 15 s bouts of cycling at 100% MAP, with recovery at 60% MAP. For the continuous low-intensity training, the warm-up was followed by 20 min of cycling at 65% MAP. Every session ended with a 10-min cool-down period at 50% of their MAP. The intensity of the continuous aerobic exercise was increased individually according to each participant’s MAP by 5% after each month, with all participants increasing to 75% MAP at the end of training for the continuous low-intensity training and 110% MAP for the HIIT training.

#### Gross motor abilities training

The GMA training protocol was adapted from a previous study [see 2 for detailed protocol]. Briefly, participants started each session with a low-intensity walking exercise on a treadmill at a self-selected pace for 10 min (max. 2.5 km/h, 1% incline). Participants then completed different exercises designed to improve coordination, balance, and agility for approximately 30 min (e.g., walking over obstacles, standing on one foot, and juggling lessons). As the intervention progressed, exercises combining multiple skills (coordination, agility, and balance) were added to increase the level of difficulty (e.g., maintaining balance on one foot and throwing a ball in a box). Participants then completed another 10 min of low-intensity walking on a treadmill. Each session concluded with five minutes of stretching and relaxation exercises.

### Statistical analyses

All data analyses were completed using IBM SPSS Statistical Software version 26. The data were screened for normality, outliers, and missing values. To evaluate the effectiveness of randomization to groups, one-way ANOVAs were carried out using each of the background measures and key outcome measures (single- and dual-task gait speed and 2-back accuracy) collected prior to the training phase. To evaluate the dual-task manipulation at baseline, paired sample t-tests were carried out on single- and dual-task gait speed and cognitive accuracy using the pre-training assessment data.

Two 2 × 3 Mixed Factorial ANOVAs were then conducted on each of the DTC scores (Cognitive, Walking) to assess change from pre-to post-training across training arms, where the within-subjects effect was Time (pre-vs. post-training) and the between-subjects effect was Training Group (EF, AE, GMA). A set of 2 × 3 ANOVAs were then conducted for each of the potential mechanisms underlying the training arms (Stroop inhibition and switching RT, CRF, and ECW) to assess the within-subjects effect of Time (pre-vs. post-training) and the between-subjects effect of Training Group (EF, AE, GMA). All follow-up analyses were Bonferroni corrected. In order to ensure that the results were not influenced by regression to the mean, all significant ANOVAs were followed up with an ANCOVA, where the covariate was the baseline score on the outcome measure found to be significant. Effect sizes were calculated as Hedges’ *g* for the t-tests and ANOVAs to account for small sample size, with the magnitude of effect being considered small (0.2 < ES ≤ 0.5), moderate (0.5 < ES ≤ 0.8), or large (ES > 0.8).

## Results

### Baseline group differences

Results revealed no significant differences (*p*s > 0.05) between groups for any of the background measures (i.e., age, sex, education, MoCA, MMSE, and TUG) or key experimental measures in the pre-training phase, suggesting that randomization was effective (Table 2).

### Baseline single- and dual-task performance

No significant differences between single- and dual-task conditions were found in performance on cognitive accuracy [*t*(32) = 1.58, *p* = 0.123], or gait speed [*t*(32) = 0.66, *p* = 0.514] at baseline. While not significant, it is notable that the range of scores was quite large, particularly for the single-task (*SD* = 19.49%) and dual-task (*SD* = 18.90%) cognitive accuracy data.

To understand the large range of cognitive task performance, we examined global cognitive status as an individual differences factor as a possible influence. Specifically, we conducted two *post-hoc* One-Way ANOVAs on single- and dual-task cognitive accuracy scores, splitting participants between low (i.e., MoCA score < 26; *n* = 14) or high cognitive status (i.e., MoCA ≥26; *n* = 18). Results showed that participants with lower MoCA scores at baseline had significantly lower cognitive accuracy under single- [*F*(1, 31) = 10.8, *p* = 0.003] and dual-task conditions [*F*(1, 31) = 10.9, *p* = 0.002], compared to participants with higher MoCA scores. Notably, there were no significant age differences between low versus high MoCA scorers.

### Training effects

Regarding walking DTCs, there was no significant effect of Time, *F*(1, 30) = 0.618, *p* = 0.438, *g* = −0.11), with DTC scores remaining similar following training (*M* = − 0.16%) compared to baseline (*M* = 0.92%). There was also no significant effect of Group (*p* = 0.406), nor a significant Time by Training Group interaction (*p* = 0.369).

**Figure 2:**
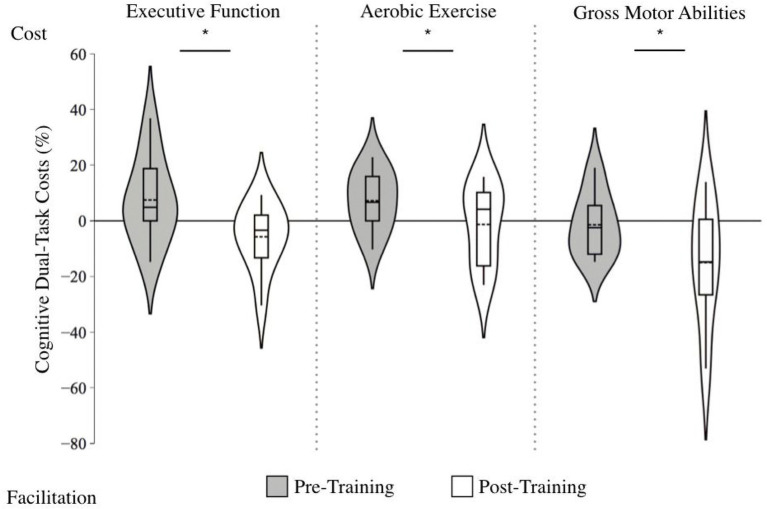
Violin plot of cognitive dual-task costs before and after training across training groups. Note: Positive scores indicate greater dual-task costs (i.e., lower 2-back accuracy during dual-tasking compared to single tasking), whereas negative scores indicate dual-task facilitation (i.e., higher 2-back accuracy during dual-task compared to single task). The length of each curve represents the range of scores, the width represents the frequency of data points in each region, the solid line within each boxplot represents the median, and the dotted line represent the mean. Following training, cognitive accuracy dual-task costs improved, regardless of the training protocol (*g* = −0.83).

For the cognitive DTCs, there was a significant main effect of Time, *F*(1, 30) = 8.82, *p* = 0.006, *g*:−0.83 (large effect size; Figure 2), indicating diminished DTCs following training, such that dual-tasking became facilitative after training (*M* = −8.03%), compared to baseline (*M* = 3.89%). There was also a significant main effect of Training Group, *F*(1, 30) = 4.36, *p* = 0.022, irrespective of Time. Follow-up pairwise comparisons revealed that the GMA group had significantly lower cognitive DTCs (*M* = −8.22%) compared to the AE group (*M* = 2.96%), *p* = 0.032. However, there was no significant interaction effect of Time and Training Group (*p* = 0.845), suggesting that all groups improved similarly. Indeed, a follow-up One-Way ANOVA comparing the change in cognitive DTCs from pre-to post-training across groups was non-significant (*p* = 0.943), and effect sizes showed similar rates of improvements across groups (*g*: EF: −0.91 [large effect], AE: −0.65 [moderate effect], GMA: −0.76 [moderate effect]). Results from the ANCOVA, where the between-subjects factor was Training Group and the covariate was baseline cognitive DTC scores, showed similar findings as our primary analysis, such that that the cognitive DTC change scores did not significantly differ across groups, *F*(2, 29) = 2.46, *p* = 0.104, suggesting that the results are not due to a regression to the mean.

**Figure 3:**
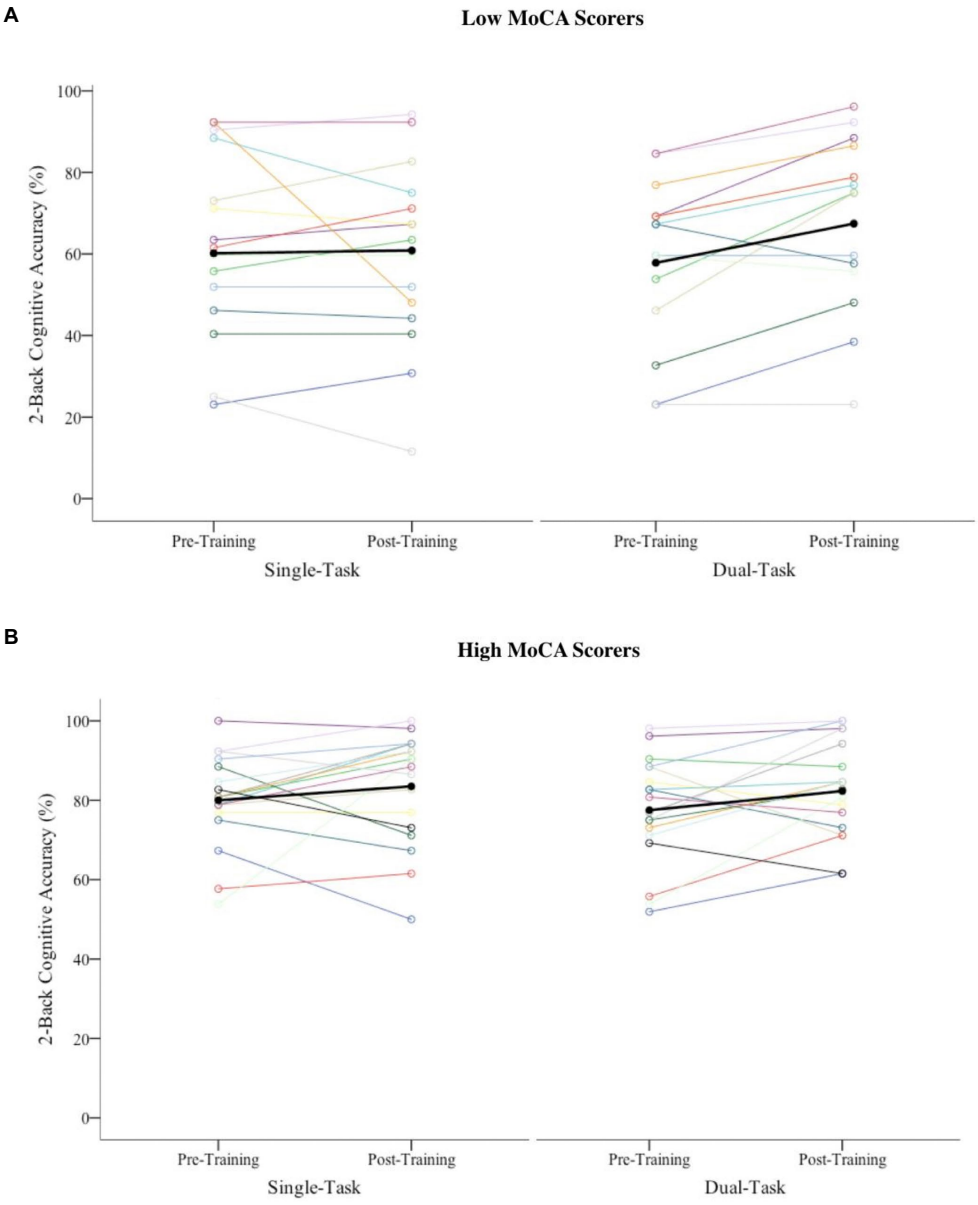
Single- and dual-task cognitive accuracy performance before and after training across participants with low versus high MoCA scores. Note: **(A)** Low baseline MoCA scorers (i.e., <26; *n* = 14), **(B)** high Low baseline MoCA scorers (i.e., ≥26; *n* = 18). Each line represents an induvial participant score (black line indicates the mean). While the high MoCA scores have the highest accuracy before and after training, there is greater improvement in the single to dual-task cost ration in low MoCA scores, leading to dual-task facilitation.

Given the unusual finding that the cognitive accuracy DTCs became better during walking (dual-task facilitation) after training, we sought to better understand this effect using *post-hoc* individual differences analyses. As mentioned, there was large variability in 2-back performance that was influenced by baseline cognitive status as measured with the MoCA. Therefore, we compared post-training cognitive DTCs across those with low (i.e., MoCA score < 26, *n* = 14) versus high cognitive status (i.e., MoCA ≥26, *n* = 18) using a One-Way ANOVA. Results showed a significant difference between groups, such that low MoCA scorers at baseline were more likely to have a dual-task facilitative effect post-training (*M* = −14.9, *SD* = 16.9), compared to high MoCA scorers, who showed negligible dual-task costs post-training (*M* = −0.17, *SD* = 11.7), *F* (1, 31) = 8.52, *p* = 0.007 (see Figure 3 for single- and dual-task scores before and after training between low vs. high MoCA scorers). To further investigate if this result was due to a regression to the mean, we conducted an ANCOVA, where the between-subjects factor was MoCA status (i.e., low vs. high scorers) and the covariate was baseline cognitive DTC scores. Results showed similar results to our initial One-Way ANOVA, such that the cognitive DTC change scores were significantly greater in the low MoCA scorers compared to the high MoCA scorers, *F*(1, 29) = 5.303, *p* = 0.029, suggesting that the results are not significantly influenced by regression to the mean.

Given the cognitive accuracy dual-task facilitation following training, we wondered whether attentional allocation to the walking task differed across participants with low versus high cognitive status. Results from the *post-hoc* One-Way ANOVA showed no significant differences in post-training walking DTCs between participants with low (*M* = 3.16 *SD* = 9.98) versus high baseline cognitive status (*M* = −2.87, *SD* = 9.88), *F* (1, 31) = 2.91, *p* = 0.10.

### Mechanisms underlying dual-task improvements

#### Executive function

**Figure 4:**
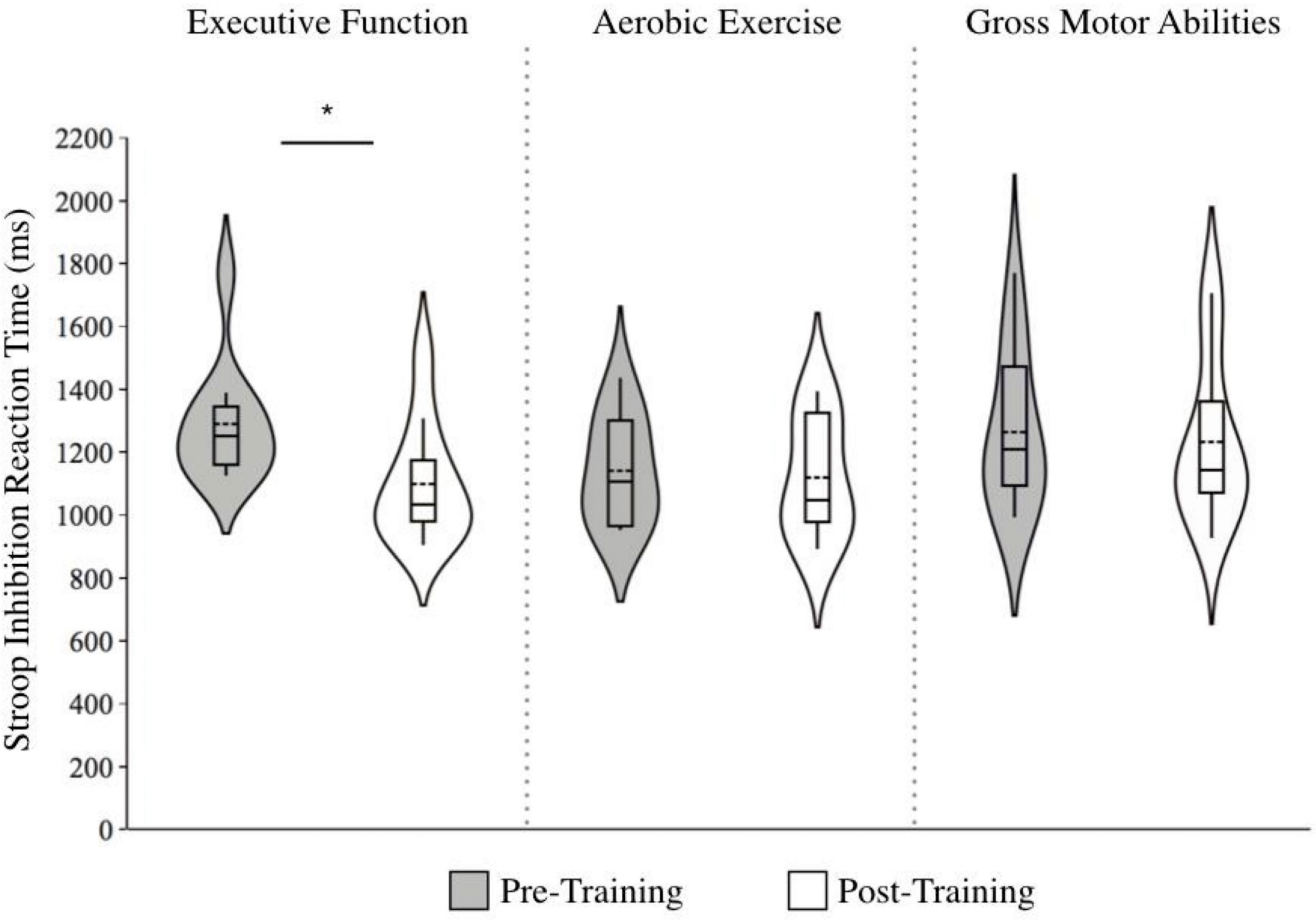
Violin plots of Stroop inhibition reaction times before and after training across training groups. Note. Lower scores indicate faster responses. The length of each curve represents the range of scores, the width represents the frequency of data points in each region, the solid line within each boxplot represents the median, and the dotted line represent the mean. There were significant improvements in reaction time for the executive function training group alone (*g* = −0.96 [large effect], ∆ = −14.8%).

**Figure 5:**
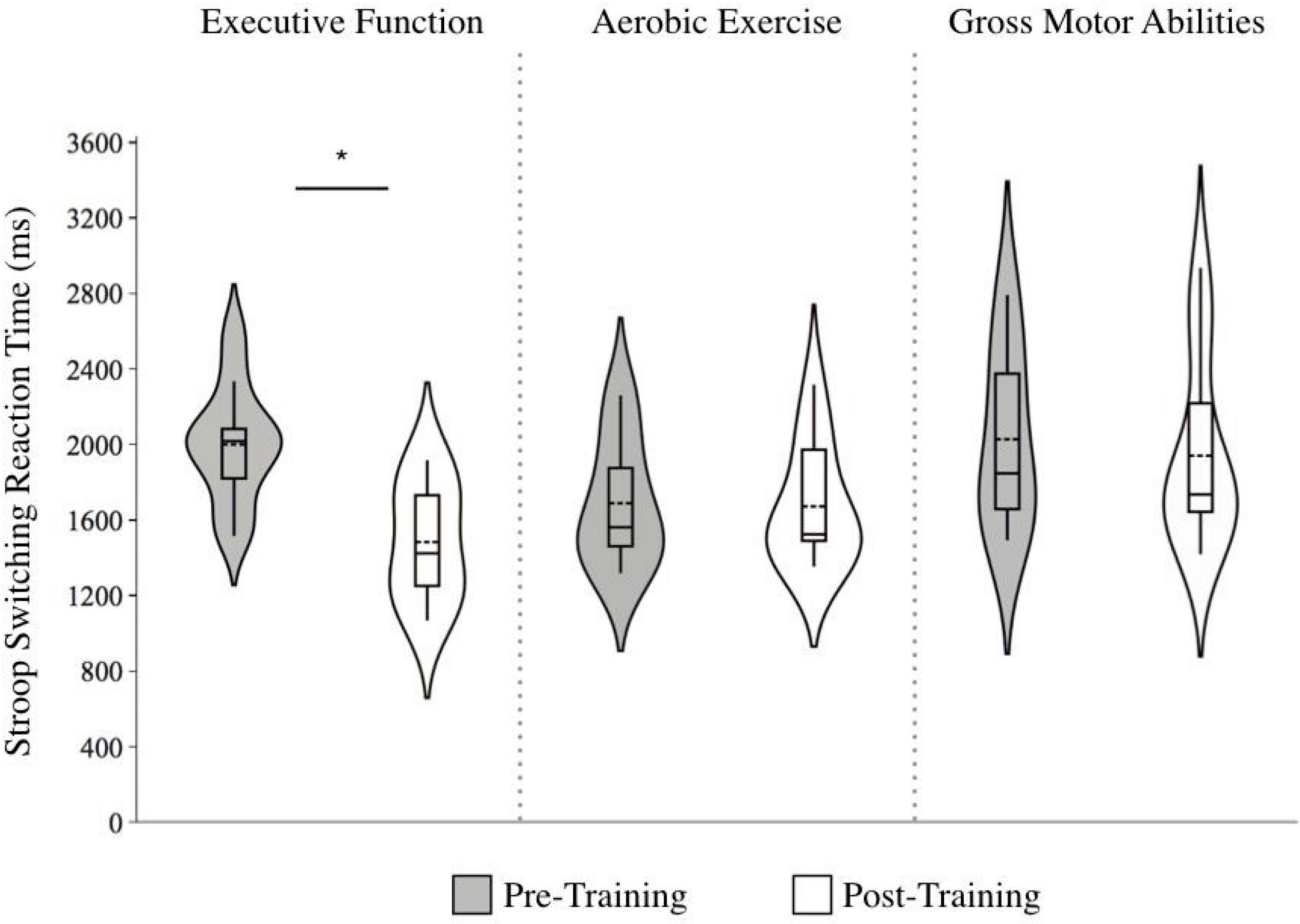
Violin plot of Stroop switching reaction times before and after training across training groups. Note: Lower scores indicate faster responses. The length of each curve represents the range of scores, the width represents the frequency of data points in each region, the solid line within each boxplot represents the median, and the dotted line represent the mean. There were significant improvements in reaction time for the executive function training group alone (*g* = −1.59 [large effect], ∆ = −25.8%).

A significant effect of Time, *F* (1, 30) = 30.3, *p* < 0.001, and a Group by Time interaction, *F* (1, 30) = 13.2, *p* = 0.001, was found for the Stroop inhibition condition, with reaction times only decreasing for the EF group; *g*: EF = −0.96 [large effect] Δ = −14.8%; AE = −0.11 [no effect], Δ = −1.90%; GMA = −0.12 [no effect], Δ = −2.49%; Figure 4. Similarly, in the switching condition, there was a significant effect of Time [*F* (1, 30) = 23.4, *p* < 0.001], as well as a Group by Time interaction, *F* (1, 30) = 13.5, *p* < 0.001, whereby only the EF group showed improvement, *F* (1, 25) = 9.18, *p* < 0.01; *g*: EF = −1.59 [large effect], Δ = −25.8%; AE = −0.05 [no effect], Δ = −0.98%; GMA = −0.25 [small effect], Δ = −4.30%; Figure 5.

#### Cardiorespiratory fitness

**Figure 6:**
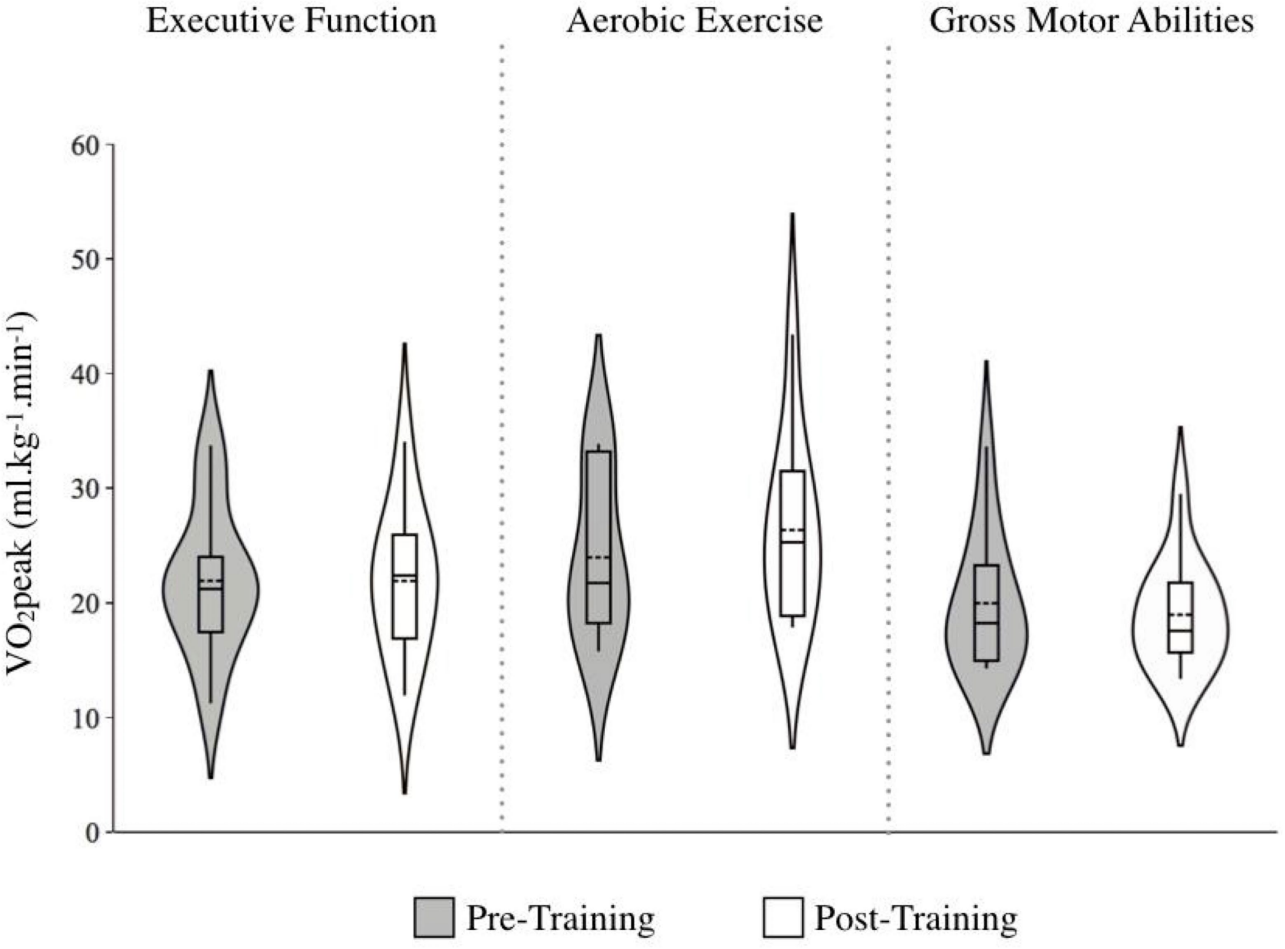
Marker of cardiorespiratory fitness (i.e., VO2peak) before and after training across training groups. Note. Higher scores indicate better cardiorespiratory fitness. The length of each curve represents the range of scores, the width represents the frequency of data points in each region, the solid line within each boxplot represents the median, and the dotted line represent the mean. While VO_2peak_ improved following aerobic exercise training (*g* = 0.31 [small effect] ∆ = 9.07%), the interaction effect only approached significant. As the aerobic exercise group had the highest VO_2peak_ levels at baseline, this may have limited the possibility for further improvement through training.

There was no significant effect of Time, *F* (1, 30) = 1.94, *p* = 0.174, though there was a Group by Time interaction that approached significance, *F* (1, 30) = 3.23, *p* = 0.054. A *post-hoc* One-Way ANOVA comparing the change in VO_2Peak_ across training groups was significant, *F* (1, 30) = 3.70, *p* = 0.037, with the AE group improving the most; AE: *g* = 0.31 [small effect] Δ = 9.07%; EF: *g* = −0.001 [no effect], Δ = −0.05%; GMA: *g* = −0.07 [no effect], Δ = −1.98%; Figure 6. Also notable is how the AE group had the highest baseline VO_2peak_ (*M* = 24.0, *SD* = 7.10), compared to EF (*M* = 21.9, *SD* = 6.48) and GMA (*M* = 19.9, *SD* = 5.84).

#### Energy cost of walking

**Figure 7:**
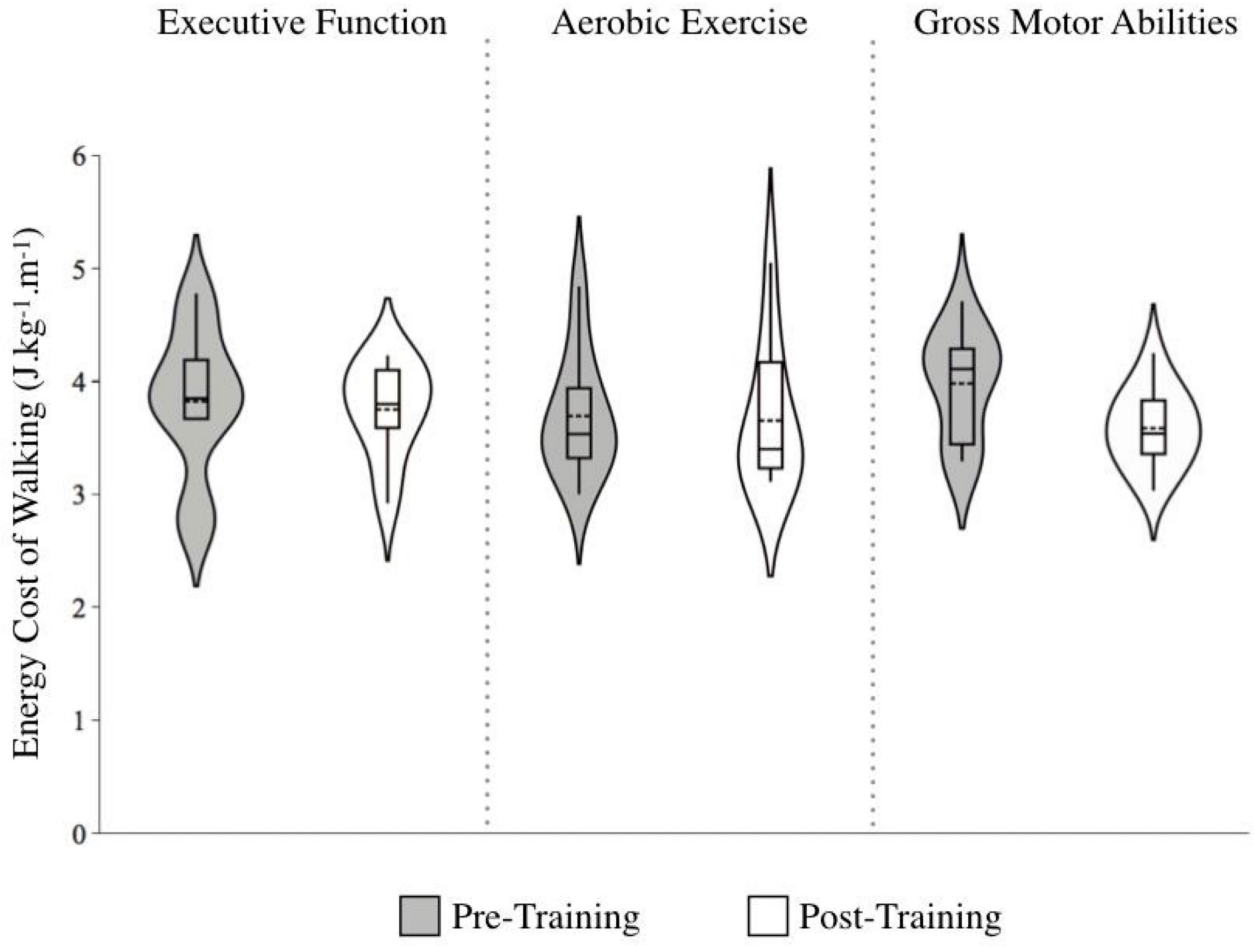
Energy cost of walking (ECW) before and after training across training groups. Note. Lower scores indicate more efficient walking. The length of each curve represents the range of scores, the width represents the frequency of data points in each region, the solid line within each boxplot represents the median, and the dotted line represent the mean. Improvements in ECW were found following gross motor abilities training (*g* = −0.94 [large effect] ∆ = −11.0%), although the interaction effect was not significant.

There was a main effect of Time that approached significance, *F* (1, 30) = 3.85, *p* = 0.059, with ECW decreasing following training. There was no significant Group by Time interaction, *F* (1, 30) = 1.86, *p* = 0.175. However, the ECW was found to decrease the most in the GMA group: *g*: GMA = −0.94 [large effect], Δ = −11.0%; EF = −0.13 [no effect], Δ = −1.93%; AE = −0.07 [no effect], Δ = −1.15%; Figure 7.

## Discussion

We aimed to evaluate the effect of cognitive training, aerobic exercise, and gross motor training on cognitive accuracy and gait speed under single- and dual-task conditions in healthy older adults. Notably, compared to pre-training levels, cognitive DTCs improved, switching to dual-task facilitation, regardless of the training modality (i.e., similar magnitudes of improvement following either cognitive or physical interventions), whereas no training effects were observed in gait speed DTCs. To better understand how various interventions have led to similar improvements in dual-task performance, we also investigated potential mechanisms specific to each training protocol. We found significant improvements in EF for participants in the cognitive training group alone, as well as larger improvements in cardiorespiratory fitness for participants in the aerobic exercise group, and greater reductions in metabolic energy demands of walking for participants in the gross motor training group. These findings are consistent with a recent study from our laboratory which had a larger sample size, showing comparable improvements in global mobility following the three interventions (Pothier et al., 2021).

### Greater improvements in cognitive vs. gait DTCs

The finding of greater improvements in cognitive DTCs compared to gait DTCs following training is consistent with our first hypothesis, which was based upon the postural prioritization hypothesis (Li et al., 2001). Specifically, since older adults tend to prioritize walking performance over cognitive accuracy during dual-tasking due to its heightened survival value, we expected there would be a greater opportunity for improvement in cognitive DTCs. Very notably, the improvements in cognitive DTCs became facilitative, meaning that cognitive accuracy became better while walking compared to while standing. However, this did not come at a decrement to gait performance.

To better understand the dual-task facilitative effect, *post-hoc* analyses were conducted, comparing dual-task performance across participants with varying levels of cognitive ability, as measured by the MoCA. First, we demonstrated that older adults with lower MoCA scores (i.e., < 26 – the clinical cut-off for mild cognitive impairment), had poorer baseline 2-back accuracy under both single and dual-task conditions. While this was expected, we did not anticipate our next finding, which was that post-training, participants with low baseline cognitive status had cognitive dual-task facilitation, whereas participants with high baseline cognitive status scores had negligible dual-task costs.

We had instead expected that older adults with high MoCA scores might have few DTCs at baseline and would be more likely to show dual-task facilitation post-training, whereas older adults with low MoCA scores might have greater costs at baseline, which would become negligible post-training. This expectation was based upon research by Wollesen and Voelcker-Rehage (2018), who demonstrated that older adults with greater physical functioning (i.e., handgrip strength) had facilitative dual-task performance in their walking speed (i.e., walked faster while doing a cognitive task than when walking alone). In contrast, older adults with lower physical functioning exhibited greater dual-task costs. Moreover, in the cognitive literature, mnemonic training has been shown to magnify individual differences based on age and baseline performance. Specifically, younger participants and those with higher baseline performance tend to have greater mnemonic gains following training (Baltes, 1987; Lövdén et al., 2012).

Although in our study participants with higher baseline MoCA scores did not have the facilitative effect as predicted, we did show that they had negligible DTCs both before and after training, which may suggest efficient complex walking behavior. For participants with lower cognitive status at baseline, an increase in cognitive resources following the intervention may have allowed greater attentional allocation to the cognitive task, leading to dual-task facilitation, while maintaining walking performance. The observed dual-task facilitation in cognitive accuracy thus appears to be driven by the proportion of older adults with lower cognitive status. This finding has direct clinical implications for older adults with low cognitive status as it demonstrates that either cognitive or physical interventions can improve cognitive efficiency during complex walking, which may reduce risk of falling and cognitive decline.

One important limitation to consider when interpreting these results is the lack of a control group, which makes it difficult to conclude whether the improvements observed in cognitive DTCs were not solely due to re-test effects. As this study followed a proof of concept design, the aim was to directly compare three well-established intervention protocols that have shown to be effective in improving cognitive or motor functioning in older adults. Indeed, EF and AE training have been shown to lead to greater improvements in executive functioning (e.g., DTCs on a computerized divided attention task, Stroop switching reaction time) compared to a placebo control group (Desjardins-Crepeau et al., 2016; Fraser et al., 2017; Bherer et al., 2021a). Single- and dual-task walking speed have also been shown to improve more following EF training compared to a wait-list control group (Verghese et al., 2010). GMA training has also been shown to improve executive control and processing speed more than a placebo control group (Voelcker-Rehage et al., 2011). These studies highlight the effectiveness of the chosen interventions for the current study, and while the current design does not deter from the possibility of re-test effects, the evidence suggests that the interventions are effective in improving cognitive or motor functioning, which may be applied when interpreting the current study findings. Additionally, an analysis to control for regression to the mean, which included baseline cognitive DTC performance as a covariate, showed similar results as our *post-hoc* analysis comparing low versus high MoCA scorers, such that there was greater improvement in cognitive DTCs in the participants with low baseline MoCA performance compared to high baseline MoCA performance. This finding suggests that our results remain significant even after controlling for regression to the mean.

Another important note to consider is that characteristics such as age and baseline neuropsychological performance may impact practice effects, with researchers showing that there are smaller practice effects in older adults compared to younger adults, as well as older adults who have poorer memory performance (Calamia et al., 2012). Given that the dual-task facilitation effect following training was primarily driven by older adults with low MoCA scores, we believe that more than 12 weeks between the pre-and post-training assessments was sufficient to reduce practice effects in this population. Nevertheless, without a control group, it is not possible to conclude whether the improvements in dual-task cognitive accuracy were not due to re-test effects, so the results should be interpreted with this in mind.

### Comparable improvement in dual-task performance across training modalities

The finding that cognitive DTCs improved regardless of training modality is consistent with our second hypothesis. While our small sample size may have left the interaction analyses underpowered, we included effect sizes to aid in interpreting our results. The effect sizes suggest similar improvement across training modalities, with the executive function training having a large effect size, and the aerobic exercise and gross motor training having moderate effect sizes. Analyses including baseline cognitive DTC scores as a covariate showed similar findings of null group differences, suggesting that the level of improvements observed across each training group was not solely due to a regression to the mean. Moreover, in a joint study involving the same training design and participants as the current study, but with a larger sample size (i.e., *n* = 78; Pothier et al., 2021), results show similar improvements in TUG walking speed regardless of training modality. We found consistent results in TUG improvements in the current sub-sample of 33 participants, suggesting that the training effects are representative of the full dataset. As such, while the small sample size is an important limitation to consider, we provide moderate evidence that may suggest similar improvements in cognitive DTCs across training modalities.

Importantly, our findings are consistent with a number of other studies which have revealed comparable improvements in dual-task walking speed and balancing (Desjardins-Crepeau et al., 2016; Fraser et al., 2017), as well as functional mobility (Pothier et al., 2021) following different combinations of cognitive and aerobic exercise training. Our findings are also in line with research demonstrating similar improvements in single- and dual-task cognitive performance following either combined high-intensity aerobic and strength training or gross motor activities (Berryman et al., 2014).

Together, our research contributes to the growing view that multiple types of interventions may be beneficial for maintaining cognitive and motor functioning in older adults. Future preference clinical trial designs may test whether having the option to participate in either cognitive or physical training might promote sustained adherence to training or could lead to increased self-efficacy in the context of cognitive-motor dual-task outcomes.

### Mechanisms underlying dual-task improvements

Given the apparent multiple routes perspective, our final aim was to better understand *how* these various interventions lead to similar improvements in dual-task performance. Executive function performance was only found to improve following the cognitive intervention, as demonstrated by reduced response times on the Stroop inhibition and switching conditions. By increasing cognitive capacity, additional resources may have been allocated to the cognitive task while dual-tasking, thereby improving performance (Li et al., 2018). Additionally, the energy demands associated with walking were found to specifically improve following gross motor activities. By increasing gait coordination and subsequently reducing the amount of energy needed to walk, dual-tasking may have been less demanding as it would require fewer physical and cognitive resources (Van Swearingen and Studenski, 2014). Finally, we hypothesized that cardiorespiratory fitness would improve the most following aerobic exercise; however, the effect size to support this was small. While the statistical testing approached significance, our results showed a 9% increase in cardiorespiratory fitness following aerobic exercise, which points to an important distinction between statistical and clinical significance. Indeed, Hawkins and Wiswell (2003) report that in older adults, VO_2max_ declines 10% per decade. Therefore, our results are clinically significant as they suggest that a relatively short-term intervention can counteract the age-normative decline in cardiorespiratory fitness. Moreover, one reason we may not have observed a statistically significant effect is due to the high baseline cardiorespiratory fitness found in the AE group, which may have limited the possibility for further improvement through training. Based upon the findings from Pothier et al. (2021), which utilized the same population and training design, but had a larger sample size, significant improvements in cardiorespiratory fitness were observed following aerobic exercise training. This therefore points to the potential limitation of our sample which had higher baseline VO_2peak_ values.

## Conclusion

The present research elucidates the impact of cognitive or physical training on the separate cognitive and motor components involved in dual-tasking and considers how different interventions may work towards improving dual-task behavior. The results suggest that regardless of training modality (EF, AE, GMA), older adults improved their cognitive performance during dual-task walking, while maintaining their gait speed. This contributes to the growing body of literature which provide evidence for a multiple routes perspective (i.e., Berryman et al., 2014; Desjardins-Crepeau et al., 2016; Fraser et al, 2017; Pothier et al., 2021). Specifically, this perspective argues that while different forms of cognitive and physical training lead to similar improvements in cognitive or motor performance, they do so through varying mechanisms. For instance, improvements in dual-task performance may have resulted from increasing executive functions following cognitive training, enhancing cardiorespiratory fitness following aerobic exercise, and reducing the metabolic energy demands following gross motor coordination training. Also notable from this research is the cognitive dual-task facilitation that was observed post-training, particularly for older adults with lower baseline cognitive status. This highlights the potential for cognitive enhancement to alter attentional allocation under complex walking conditions in individuals with lower cognitive ability. Our research findings are important given the functional implications of reduced dual-task performance in old age (e.g., increased risk of falls, cognitive impairment). However, due to the limitations of this study, including a small sample size and lack of a control group, future research is needed to substantiate the current study findings, ideally in a larger randomized control trial.

## Data availability statement

The raw data supporting the conclusions of this article will be made available by the authors, without undue reservation.

## Ethics statement

The studies involving human participants were reviewed and approved by Institut Universitaire Gériatrie de Montreal ethical research committee and Concordia University’s Human Research Ethics Committee. The patients/participants provided their written informed consent to participate in this study.

## Author contributions

RD: conceptualization, data curation, methodology, validation, formal analysis, and writing—original draft—reviewing and editing. LBh: conceptualization, data curation, funding acquisition, investigation, methodology, project administration, validation, and Writing—reviewing and editing. KP: conceptualization, data curation, methodology, project administration, validation, and writing—reviewing and editing. TVr: conceptualization, data curation, formal analysis, methodology, validation, and writing—reviewing and editing. BI: conceptualization, data curation, methodology, project administration, validation, and writing—reviewing. NB: conceptualization, data curation, funding acquisition, methodology, validation, and writing—reviewing and editing. ML: conceptualization, data curation, formal analysis, methodology, validation, and writing—reviewing. TVi: data curation, methodology, project administration, validation, and writing—reviewing. AK: conceptualization, funding acquisition, validation, and writing—reviewing and editing. AN: conceptualization, data curation, funding acquisition, investigation, validation, and writing—reviewing. TVu: conceptualization, data curation, funding acquisition, investigation, validation, and writing—reviewing. LBo: conceptualization, funding acquisition, validation, and writing—reviewing. KL: conceptualization, data curation, formal analysis, funding acquisition, investigation, methodology, validation, and writing—reviewing and editing. All authors contributed to the article and approved the submitted version.

## Funding

This material is based upon work supported by the Canadian Institute of Health Research under grant no. 136859.

## Conflict of interest

The authors declare that the research was conducted in the absence of any commercial or financial relationships that could be construed as a potential conflict of interest.

## Publisher’s note

All claims expressed in this article are solely those of the authors and do not necessarily represent those of their affiliated organizations, or those of the publisher, the editors and the reviewers. Any product that may be evaluated in this article, or claim that may be made by its manufacturer, is not guaranteed or endorsed by the publisher.
